# Detection and Characterization of Human Pegivirus 2, Vietnam

**DOI:** 10.3201/eid2411.180668

**Published:** 2018-11

**Authors:** Nguyen To Anh, Nguyen Thi Thu Hong, Le Nguyen Truc Nhu, Tran Tan Thanh, Catherine Anscombe, Le Ngoc Chau, Tran Thi Thanh Thanh, Chuen-Yen Lau, Direk Limmathurotsakul, Nguyen Van Vinh Chau, H. Rogier van Doorn, Xutao Deng, Motiur Rahman, Eric Delwart, Thuy Le, Guy Thwaites, Le Van Tan, for the Southeast Asia Infectious Disease Clinical Research Network

**Affiliations:** Oxford University, Ho Chi Minh City, Vietnam (N.T. Anh, N.T.T. Hong, L.N.T. Nhu, T.T. Thanh, C. Anscombe, L.N. Chau, T.T.T. Thanh, H.R. van Doorn, M. Rahman, T. Le, G. Thwaites, L.V. Tan);; University of Oxford, Oxford, UK (C. Anscombe, D. Limmathurotsakul, H.R. van Doorn, G. Thwaites);; National Institutes of Health, Bethesda, Maryland, USA (C.-Y. Lau);; Mahidol Oxford Tropical Research Unit, Bangkok, Thailand (D. Limmathurotsakul);; Hospital for Tropical Diseases, Ho Chi Minh City, Vietnam (N.V.V. Chau);; Blood Systems Research Institute, San Francisco, California, USA (X. Deng, E. Delwart);; University of California, San Francisco (X. Deng, E. Delwart)

**Keywords:** human pegivirus 2, human hepegivirus 1, Vietnam, HCV, HIV, hepatitis and sepsis, viruses

## Abstract

We report human pegivirus 2 (HPgV-2) infection in Vietnam. We detected HPgV-2 in some patients with hepatitis C virus/HIV co-infection but not in patients with HIV or hepatitis A, B, or C virus infection, nor in healthy controls. HPgV-2 strains in Vietnam are phylogenetically related to global strains.

Human pegivirus 2 (HPgV-2), also known as human hepegivirus 1, is a recently discovered bloodborne flavivirus (*1*,*2*). Existing evidence suggests that HPgV-2 is more frequently detected in patients with hepatitis C virus (HCV) infection, particularly HCV and HIV co-infection, although detection rates vary between studies and patient groups. In the United States, HPgV-2 was detected in 1.2% (12/983) of patients with active HCV infections (*1*), whereas in China, the reported detection rates of HPgV-2 RNA were 0.29% (7/2440) among HCV monoinfected patients and from 3% (8/270) to 5.7% (4/70) among HCV/HIV co-infected patients (*3*,*4*). HPgV-2 RNA was detected in 10.9% (17/156) of injection drug users in the United States, and there was a strong association between HPgV-2 and other infections such as HCV and SEN virus D (*5*).

Given the high burden of HCV and HIV infections worldwide and the potential clinical significance of HPgV-2, we investigated the geographic distribution and genetic diversity of this virus to help prioritize the development and implementation of appropriate intervention strategies. The studies were approved by the corresponding institutional review boards of the local hospitals in Vietnam where patients were enrolled and the Oxford Tropical Research Ethics Committee. We obtained written informed consent from each participant or from the participant’s parent or legal guardian.

## The Study

Patient information and clinical samples were derived from a multilocation observational study designed to evaluate the causes of community-acquired infection in Southeast Asia (*6*). We included all 493 samples (384 plasma, 92 pooled nasal and throat swabs, 10 stool, and 7 cerebrospinal fluid [CSF]) from 386 patients in Vietnam with community-acquired infection of unknown origin after extensive diagnostic workup for viral metagenomic analysis (*7*).

Analysis of metagenomic data revealed that, in 1 plasma sample, of 98,344 obtained reads, 5,342 reads were of HCV sequences, 430 of HIV sequences, and 273 of HPgV-2 sequences; we confirmed all reads by corresponding virus-specific reverse transcription PCR (RT-PCR). HPgV-2 sequence screening and HPgV-2 RT-PCR testing did not detect HPgV-2 in any of the remaining samples of the patients included in metagenomic analysis.

To explore the prevalence of HPgV-2 in HCV-infected patients in Vietnam, we used a reference-based mapping strategy to screen for HPgV-2 sequences in additional viral metagenomic datasets (Table 1). We detected HPgV-2 sequences in 5/79 HIV/HCV co-infected patients who participated in a trial evaluating the hepatic safety of raltegravir/efavirenz-based therapies in antiretroviral-naive HIV-infected subjects co-infected with HCV. We did not detect HPgV-2 sequences in 394 HCV-infected patients with clinically diagnosed hepatitis who participated in molecular epidemiologic studies of hepatitis viruses (Table 1). 

**Table 1:** Samples and viral metagenomic datasets used in screening for human pegivirus, and screening results, Vietnam*

Infection	No. persons	Screening approach	No. positive for HPgV-2	Enrollment period	Setting
Hepatitis C virus and HIV co-infection	79	HPgV-2–specific PCR and reference-based mapping of obtained viral metagenomics data	5	2010–2013	Hospital for Tropical Diseases, Ho Chi Minh City
HIV monoinfection	78	HPgV-2–specific PCR	0	2010–2013	Hospital for Tropical Diseases, Ho Chi Minh City
None (healthy volunteers)	80	HPgV-2–specific PCR	0	2010–2013	Hospital for Tropical Diseases, Ho Chi Minh City
Hepatitis A virus	71	HPgV-2–specific PCR	0	2012–2014	Hospital for Tropical Diseases, Ho Chi Minh City
Hepatitis B virus	103	HPgV-2–specific PCR	0	2012–2016	Hospital for Tropical Diseases, Ho Chi Minh City; Dong Thap General Hospital, Dong Thap; Khanh Hoa Provincial Hospital, Nha Trang; Dac Lac Provincial Hospital, Dac Lac; Hue National Hospital, Hue
Hepatitis C virus†	394	Reference-based mapping of obtained viral metagenomics data	0	2012–2016	Hospital for Tropical Diseases, Ho Chi Minh City; Dong Thap General Hospital, Dong Thap; Khanh Hoa Provincial Hospital, Nha Trang; Dac Lac Provincial Hospital, Dac Lac; Hue National Hospital, Hue

*HPgV-2, human pegivirus. †Whole-genome sequences of hepatitis C virus were obtained using a viral metagenomics approach (*7*). The resulting metagenomics datasets were then subjected to a reference-based mapping approach to search for the presence of HPgV-2 sequences.

We subsequently confirmed the result of this reference-mapping approach by HPgV-2 multiplex RT-PCR (*8*) testing of the extracted RNA from original samples. We conducted multiplex RT-PCR screening for HPgV-2 RNA in plasma samples of matched controls (78 HIV-infected patients and 80 healthy volunteers) of the 79 HCV/HIV co-infected patients; we found no evidence of HPgV-2 (Table 1). In addition, we did not detect HPgV-2 RNA in any plasma samples from patients with HAV (n = 71) and HBV (n = 103) infection (Table 1).

HPgV-2 RNA was detectable for <18 months in 3/5 patients with HCV/HIV co-infection (Table 2). We did not detect HPgV-2 RNA in the available follow-up serum sample collected 14 days after enrollment from the patient with community-acquired infection (Table 2).

**Table 2:** Demographic and clinical features of 6 men with human pegivirus infection, Vietnam*

Pt no.	Pt age, y	Time point, mo	HCV RNA+	HPgV-2 RNA+	Total bilirubin, µmol/L	Direct bilirubin, µmol/L	AST, UI/L	ALT, UI/L	CD4 count, cells/µL	HIV RNA, × 10^3^ copies/µL	AFP, mg/mL	FibroScan result, kPa	Symptoms	
1	29	NA	NA	NA	NA	NA	NA	NA	NA	NA	NA	NA	NA	
2	47	0	Y	Y	9.8	0.7	30	24	331	120	1.7	11.8		
		6	Y	Y	4.7	1.6	81	83	518	0.07	2.3	NA		
		12	Y	Y	6.9	3.4	55	61	364	0.04	2.6	11.8	Hepatitis	
		18	Y	Y	4.8	2.8	37	40	428	UND	2.14	6.1	Hepatomegaly	
3	32	0	Y	Y	4.7	3.4	39	10	288	0.198	0.999	6.5		
		6	Y	Y	12.8	4.7	50	19	510	0.04	1.68	NA		
		12	Y	Y	9.5	5.3	63	25	622	UND	1.88	6.2	Liver fibrosis, hepatomegaly	
		18	Y	Y	7.6	3.8	42	23	622	UND	1.53	7.2	Hepatitis	
4	35	0	Y	Y	7.8	4.9	67	55	290	61.1	2.96	6.4		
		6	Y	Y	10.7	6.3	77	80	411	UND	3.1	NA		
		12	Y	Y	8.8	3.9	76	72	337	UND	4	8.5	Homogeneous hepatomegaly	
		18	Y	Y	13	6.3	108	129	455	UND	4.1	8.1	Splenomegaly, liver fibrosis	
5	34	0	Y	Y	4.3	2.8	33	43	291	70.2	3.67	6.1		
		6	N	Y	6.5	2.1	35	43	287	UND	3.83	NA		
		12	N	N	5.4	2.6	33	40	484	UND	4.48	4.5		
		18	N	N	6.6	2.6	73	85	546	UND	3.9	3		
6	31	0	Y	Y	4.5	2.4	52.2	36.5	295	96.8	12.7	22.8		
		6	Y	Y	17.1	12.9	64	62	579	UND	16.74	NA		
		12	Y	N	12.3	4.3	114	121	711	UND	46.3	26.3	Mild liver fibrosis, mild splenomegaly	
		18	Y	N	10.6	4.9	82	89	816	UND	61.01	NA	Hepatomegaly, splenomegaly	

*Age is patient’s age at diagnosis; time point is the month at which follow-up visit was conducted; 0 was the baseline examination. ALT, alanine aminotransferase; AS, aspartate aminotransferase; NA, not available; Pt, patient; UND, undetectable.  †Patient 1 belongs to the community-acquired infection cohort.

All 5 HCV/HIV co-infected patients had CD4 counts >200 cells/µL at baseline and at 6-, 12-, and 18-month follow-up (Table 2), but none received specific anti-HCV drugs, which was attributed to drug unavailability or unaffordability during the study period. During follow-up, hepatitis and splenic abnormalities were detected in 4/5 patients, which were likely attributable to HCV infection (Table 2). The patient with community-acquired infection was recorded as surviving to 28 days of follow-up (Table 2).

Using deep sequencing and a combination of overlapping PCRs and Sanger sequencing of PCR amplicons (primer sequences available upon request), we obtained 5 nearly complete genomes (coverage of >92%) and another partial genome (coverage of ≈69.1%). Pairwise comparison of HPgV-2 coding regions obtained in this study and previously reported HPgV-2 sequences showed overall sequence identities at the nucleotide level of >94.6% and at the amino acid level of >95.3% (data not shown). Phylogenetic analyses revealed a tight cluster between viruses from Vietnam and global strains (Figure). We submitted the HPgV-2 sequences we generated to GenBank (accession nos. MH194408–13).

**Figure:**
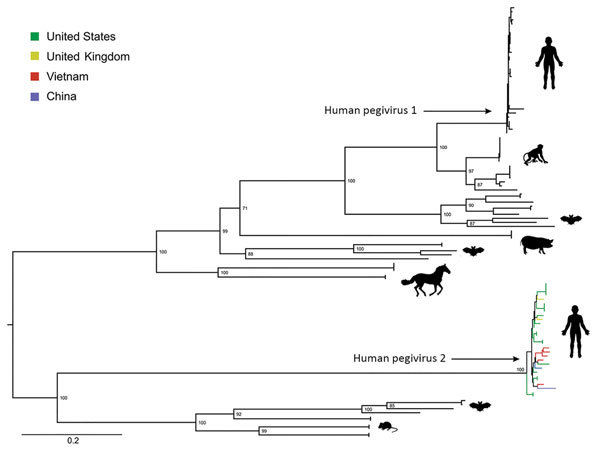
Maximum-likelihood phylogenetic tree of amino acid sequences of coding sequences of human pegivirus 2 strains from Vietnam compared with global strains and other pegiviruses. We used the general matrix with empirical amino acid frequencies, a gamma distribution of 4 rates, and invariant sites, as suggested by IQ TREE (http://www.iqtree.org), to reconstruct the phylogenetic trees. We assessed support for individual nodes using a bootstrap procedure of 10,000 replicates. Scale bar indicates amino acid substitutions per site.

Of the 5 HPgV-2 genome sequences we recovered, we generated 2 by deep sequencing. The results were above the proposed sequencing-depth threshold of >5 for sequences generated by next-generation sequencing (*9*) and sufficient for intrahost diversity investigation. One sequence we generated had mean coverage of 2,049 (range 12–9,912), with a total of 26 (10 [38%] nonsynonymous) positions carrying minor variations detected in the corresponding dataset (data not shown). For the other sequence, mean coverage was 32,531 (range 13–138,383), with a total of 37 (13 [35%] nonsynonymous) positions carrying minor variations in its dataset (data not shown).

## Conclusions

We report the detection and genetic characterization of HPgV-2 in Vietnam and describe the observed demographic and clinical characteristics of patients with HPgV-2 infection. Together with reports from China, Iran, and the United States (*1*–*4*,*8**,**10*), our findings further emphasize the strong association between HPgV-2 and HCV, especially HCV/HIV co-infection. The absence of HPgV-2 in 394 HCV-infected patients may have been attributed to the small sample size and the fact that the reported prevalence of HIV among HCV-infected patients was <6.5% (*11*,*12*). Of note, HPgV-2 was detected in only 0.29% of HCV-monoinfected patients in China.

Previous reports showed that HPgV-2 viremia can be transient or persistent. Likewise, in our study, HPgV-2 RNA became undetectable after 14 days in a HCV/HIV co-infected patient with community-acquired infection of unknown origin, but remained detectable in other HCV/HIV co-infected patients through up to 18 months of follow-up.

The pathogenic potential of HPgV-2 remains unknown. Its role in HCV/HIV co-infection and response to treatment warrants further research, given its low detection rates in blood donors in the United States and China (*1*,*3*) and its absence in healthy persons (this study) but close association with HCV/HIV co-infection.

In the era of sequence-based virus discovery, a key question is whether the detected genome represents live virus or a non–replication competent genome. Addressing this question would require recovery of virus in cell culture. However, our detection of minor variations across 2 HPgV-2 genomes suggests that viral replication had occurred in the infected patients. Phylogenetically, the close relatedness between HPgV-2 strains from Vietnam and global strains suggests HPgV-2 has a wide geographic distribution.

Our study has some limitations. First, we only retrospectively tested available archived samples without formal sample size estimation, which may have explained the absence of HPgV-2 in the remaining 394 HCV patients. Second, we did not employ a serologic assay to screen for HPgV-2 antibodies in patients’ plasma. Third, we used only multiplex PCR with primers based on a limited number of available HPgV-2 sequences. Therefore, we may have missed genetically diverse HPgV-2 strains, and we may have underestimated the prevalence of HPgV-2 infections in Vietnam.

Our findings contribute expanded data about geographic distribution, demographics, and genetic diversity of HPgV-2. Because HCV and HIV infections are global public health issues, the extent to which HPgV-2 interacts with HCV and HIV in co-infected patients and the possible clinical consequences warrant further research.
